# Prior Vaccination Exceeds Prior Infection in Eliciting Innate and Humoral Immune Responses in Omicron Infected Outpatients

**DOI:** 10.3389/fimmu.2022.916686

**Published:** 2022-06-15

**Authors:** Hye Kyung Lee, Ludwig Knabl, Mary Walter, Ludwig Knabl, Yuhai Dai, Magdalena Füßl, Yasemin Caf, Claudia Jeller, Philipp Knabl, Martina Obermoser, Christof Baurecht, Norbert Kaiser, August Zabernigg, Gernot M. Wurdinger, Priscilla A. Furth, Lothar Hennighausen

**Affiliations:** ^1^National Institute of Diabetes, Digestive and Kidney Diseases, National Institutes of Health, Bethesda, MD, United States; ^2^TyrolPath Obrist Brunhuber GmbH, Zams, Austria; ^3^Clinical Core, National Institute of Diabetes, Digestive and Kidney Diseases, National Institutes of Health, Bethesda, MD, United States; ^4^Division of Internal Medicine, Krankenhaus St. Vinzenz, Zams, Austria; ^5^Division of Internal Medicine, Krankenhaus St. Johann, St. Johann, Austria; ^6^Division of Internal Medicine, Krankenhaus Kufstein, Kufstein, Austria; ^7^Departments of Oncology and Medicine, Georgetown University, Washington, DC, United States

**Keywords:** SARS-CoV-2 omicron infection, prior COVID-19 infection, antibody responses, immune transcriptome, COVID-19 alpha infection, interferon response, anti-viral response

## Abstract

Antibody response following Omicron infection is reported to be less robust than that to other variants. Here we investigated how prior vaccination and/or prior infection modulates that response. Disease severity, antibody responses and immune transcriptomes were characterized in four groups of Omicron-infected outpatients (n=83): unvaccinated/no prior infection, vaccinated/no prior infection, unvaccinated/prior infection and vaccinated/prior infection. The percentage of patients with asymptomatic or mild disease was highest in the vaccinated/no prior infection group (87%) and lowest in the unvaccinated/no prior infection group (47%). Significant anti-Omicron spike antibody levels and neutralizing activity were detected in the vaccinated group immediately after infection but were not present in the unvaccinated/no prior infection group. Within two weeks, antibody levels against Omicron, increased. Omicron neutralizing activity in the vaccinated group exceeded that of the prior infection group. No increase in neutralizing activity in the unvaccinated/no prior infection group was seen. The unvaccinated/prior infection group showed an intermediate response. We then investigated the early transcriptomic response following Omicron infection in these outpatient populations and compared it to that found in unvaccinated hospitalized patients with Alpha infection. Omicron infected patients showed a gradient of transcriptional response dependent upon whether or not they were previously vaccinated or infected. Vaccinated patients showed a significantly blunted interferon response as compared to both unvaccinated Omicron infected outpatients and unvaccinated Alpha infected hospitalized patients typified by the response of specific gene classes such as OAS and IFIT that control anti-viral responses and IFI27, a predictor of disease outcome.

## Introduction

The highly transmissible Omicron (B.1.1.529) variant is less susceptible to neutralizing antibodies elicited by previous vaccination or other variant infection (1–4), thus resulting in a continuation of the COVID-19 pandemic. While recent studies have investigated the antibody response to breakthrough infections (5, 6), there is a knowledge gap about the humoral and genomic immune response to Omicron infection in outpatients that had been vaccinated, previously infected with another SARS-CoV-2 variant or both. Specifically, the impact of previous infection as compared to vaccination in the generation of anti-Omicron spike antibodies upon Omicron infection has yet to be determined.

While Omicron infections generally result in a more moderate symptomology compared to other variants, the genomic immune response in this patient population has not been investigated. Transcriptome studies on hospitalized patients infected with the Alpha (7) or Beta (8) variants have revealed the activation of interferon pathway genes with an emphasis of the JAK/STAT pathway. While the interferon response in severely ill patients has been reported, it is not clear if Omicron patients with a generally lighter symptomology have a different immune transcriptome which can be further modulated by vaccination and prior infection.

To address these questions, we investigate the transcriptional and humoral immune response in 83 outpatients with documented Omicron infection. Thirty-four had no prior infection and were not vaccinated, 23 persons had been vaccinated and boosted with the BNT162b2 mRNA vaccine (Pfizer–BioNTech), 19 had a history of prior infection by other SARS-CoV-2 variants and 7 had been vaccinated and had a prior infection. This study permitted an understanding of how vaccination and prior infection impact the immune response to Omicron infection and we identified a gradient response that corresponds to the humoral profile.

## Results

### Study Design

**Figure 1:**
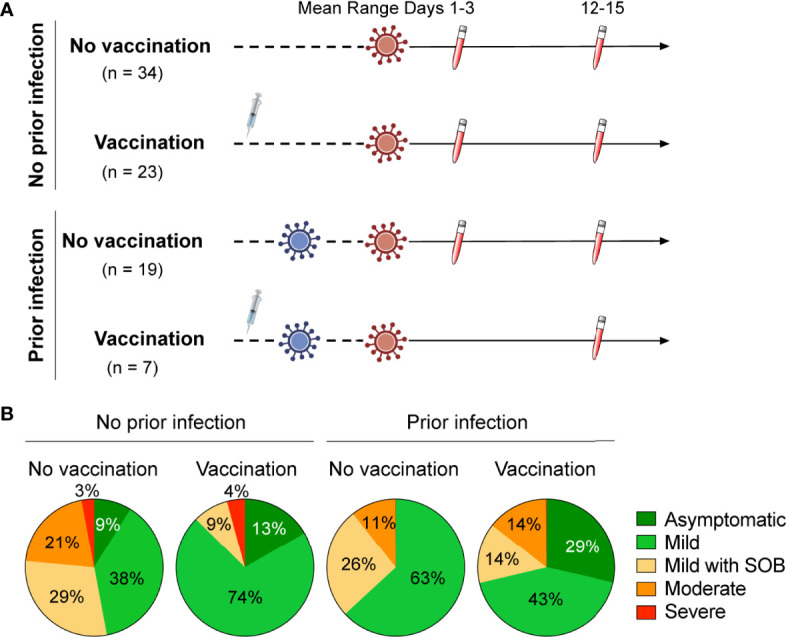
Study design and outpatient symptomology. **(A)** Schematic presentation of the experimental workflow. All 83 study subjects were infected by the SARS-CoV-2 Omicron variant with or without prior infection with another SARS-CoV-2 variant. Four groups were studied: no vaccination/no prior infection (n=34), vaccination/no prior infection (n=23), no vaccination/prior infection (n=19) and vaccination/prior infection (n=7) (**Table 1**). Blood was collected from the outpatients from three groups at two timepoints and from the fourth group at one time point after testing PCR positive. The range of the means of the different groups are shown. **(B)** Symptomology of the study cohorts. SOB, shortness of breath.

The first case of Omicron infection in Austria was reported at the end of November 2021 (9) and recruitment of outpatients took place between December 2021 and March 2022. Here, we investigate the humoral and transcriptional immune response in 83 persons with documented Omicron infection. Four groups were studied: no vaccination/no prior infection (n=34), vaccination/no prior infection (n=23), no vaccination/prior infection (n=19) and vaccination/prior infection (n=7) (**Figure 1A**; **Table 1**). Demographic and clinical characteristics of the study population are provided in **Table 1**. The transcriptional response of the first three groups was investigated at days 1-3 following initial SARS-CoV-2 RT-PCR test and the humoral response at two timepoints (range of group means days 1-3 and 12-15 following initial SARS-CoV-2 RT-PCR test). The humoral response for the fourth group (vaccination/prior infection) was measured at range of group means days 12-15 following initial SARS-CoV-2 RT-PCR test for comparison. Due to recruitment challenges and to the low numbers of these people in the community studied, only samples for the later timepoint were available for that group. Transcriptional studies were limited to days 1-3, the point of highest immune transcriptional response. The percentage of patients with asymptomatic or mild disease without respiratory symptoms was highest in the vaccination/no prior infection group (87%), followed by the no vaccination/prior infection group (63% with mild disease, no asymptomatic), the no vaccination/no prior infection (47% with either mild disease or asymptomatic) group and the vaccination/prior infection (72% with either mild disease or asymptomatic) group (**Figure 1B**). Moderate and severe cases accounted for 24% in the no vaccination/no prior infection group but only 4% of the vaccinated group, 11% in the no vaccination/prior infection group and 14% in the vaccinated/prior infection group (**Figure 1B**).

**Table 1:** Characteristics of Omicron study population.

	No prior infection		Prior infection		
	No vaccination	Vaccination	No vaccination	Vaccination	Chi-Square
**Age** (years), mean (range)	45 (9-83)	38 (17-82)	33 (10-63)	36 (24-52)	9.07
**Gender** Female	26 (76%)	13 (57%)	10 (53%)	5 (71%)	0.87
Male	8 (24%)	10 (43%)	9 (47%)	2 (29%)	2.78
**Medical condition**	16 (47%)	8 (35%)	5 (26%)	1 (14%)	1.98
Auto-immune Disease	1	2	3	0	
Chronic Heart Disease	1	2	0	0	
Chronic Pulmonary Disease	2	1	0	0	
Dementia	1	0	0	0	
Diabetes	1	0	0	0	
Gout	1	0	0	0	
Hypertension	3	1	0	0	
Hypothyroid	3	0	2	0	
Kidney Disease	1	0	0	0	
Multiple Sclerosis	1	2	0	0	
S/P Cancer	1	0	0	1	
**COVID-19 vaccine history**	0	23	0	7	
3 doses	0	10	0	2	
2 doses	0	13	0	5	
**Days from last vaccine dose to positive PCR test, mean (range)**	N/A	111 (1-294)	N/A	126 (34-303)	
**Days from prior infection dose to positive PCR test, mean (range)**	N/A	N/A	98 (21-289)	124 (60-172)	
**Days from positive PCR test to sampling, mean** **(range)** 1st sampling	2 (0-5)	1 (0-3)	3 (0-5)	0	
2nd sampling	13 (6-17)	13 (6-14)	12 (6-18)	15 (6-19)	

N/A, not applicable.

### Vaccination Is Superior to Prior Infection in Preparing the Immune Response to Omicron

**Figure 2:**
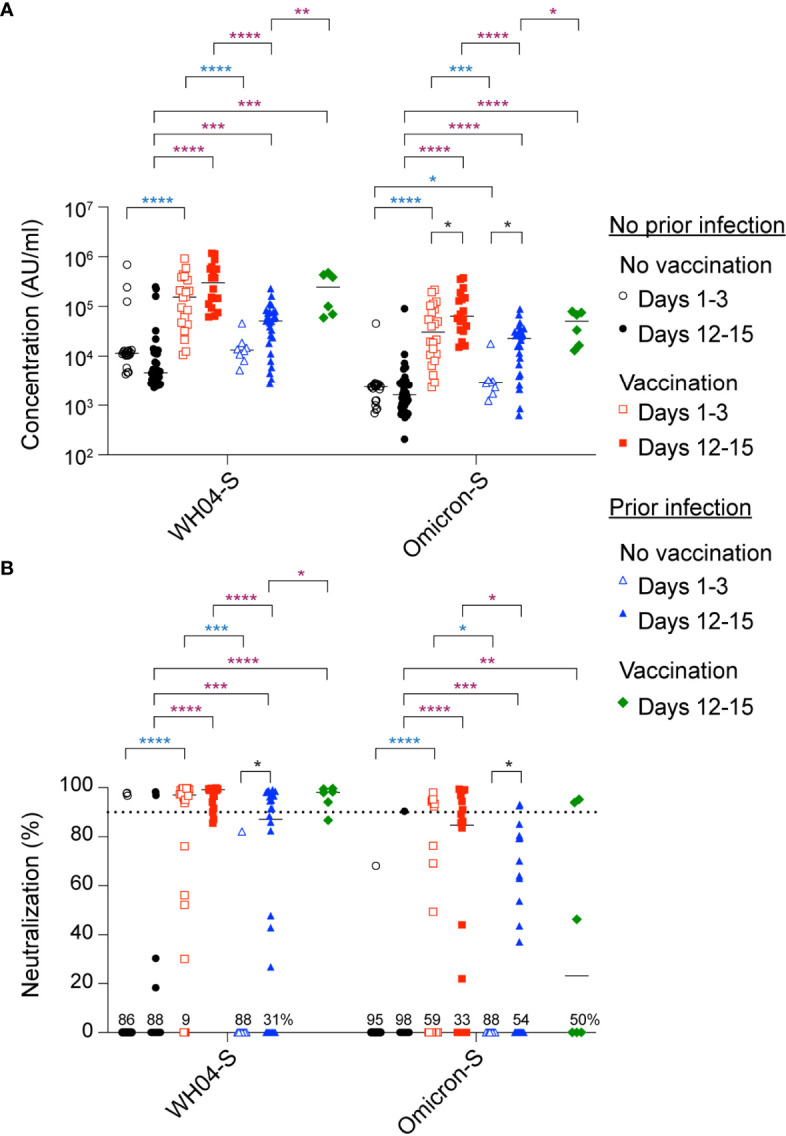
Antibody analysis. **(A)** Plasma IgG antibody binding the SARS-CoV-2 RBD (spike) from the ancestral and Omicron strains in the unvaccinated and vaccinated Omicron patients without or with prior infection experience. **(B)** Neutralization response to virus spike protein of the ancestral and Omicron variants. *p*-value between two groups is from one-tailed Mann-Whitney t-test. Black asterisks indicate significance between days within the same group, blue asterisks indicate significance between groups (range of group means days1-3), purple asterisks indicate significance between groups (range of group means days 12-15). Percentage of samples with zero neutralization activity for each group are indicated over the X axis. *p < 0.05, **p < 0.01, ***p < 0.001, ****P < 0.0001. Line at median, dotted line at 90%.

First, we measured circulating anti-spike antibody levels in serum samples obtained from the Omicron outpatients within the first three days (days 1-3) following initial SARS-CoV-2 RT-PCR test (**Figure 2**). The anti-Omicron spike IgG levels were highest in the vaccinated patients without prior infection and approximately 10-fold lower in the unvaccinated patients with and without prior infection (**Figure 2A**). An equivalent pattern was obtained for the ancestral strain (**Figure 2A**) and other variants (**Figure S1A**). A significant increase of anti-Omicron spike, but not anti-ancestral spike, antibodies was observed in the vaccinated and the previously infected group within 12-15 days following Omicron infection (**Figure 2A**). Antibody levels did not increase in the naïve group. These findings are mirrored by anti-spike antibody levels from other variants (**Figure S1A**).

At this point in the pandemic, a critical question is whether prior BNT162b2 vaccination can prompt development of neutralizing antibodies in Omicron infected individuals. Here, we assessed neutralization capacity using the angiotensin-converting enzyme 2 (ACE2) binding inhibition assay, against the Omicron spike protein and those from other variants. We measured neutralization within three days following initial SARS-CoV-2 RT-PCR test (Days 1-3) and after 12-15 days (**Figure 2B**). Significant (approximately 11-fold higher in the vaccinated group compared to the no vaccinated group) Omicron neutralizing activity was seen in the vaccinated group, which further increased after 12-15 days following initial SARS-CoV-2 RT-PCR test. Significant neutralizing activity was detected in the previously infected group, with diverse increase, but less than the vaccinated group, at day 12-15 following initial SARS-CoV-2 RT-PCR test. These findings parallel those seen for other variants (**Figure S1B**).

### Vaccination but Not Prior Infection Blunts Interferon Responses Elicited by Omicron Infection

**Figure 3:**
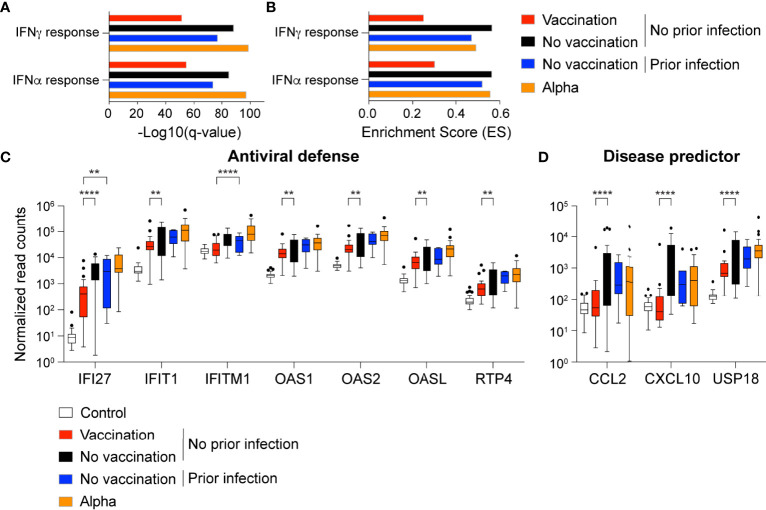
Immune transcriptomes following Omicron infection. **(A, B)** Gene categories expressed at significantly higher levels in unvaccinated and vaccinated Omicron patients without prior SARS-CoV-2 infection, unvaccinated Omicron patients with prior SARS-CoV-2 infection, and a reference group of patients infected by the SARS-CoV-2 Alpha variant were significantly enriched in interferon-activated and inflammatory pathways. X-axis denotes statistical significance as measured by minus logarithm of FDR q-values **(A)** and enrichment score (ES) **(B)**, respectively. Y-axis ranked the terms by q values **(A)** or enrichment score **(B)**. **(C, D)** Bar plots with the normalized read counts to mRNA levels of fifteen innate immune response genes of the 23 genes that are significantly induced in all cohorts and higher in the ‘no vaccination group’ compared to ‘vaccination group’ **(C)** and seven that are significantly induced in the ‘no vaccination’ group, but not ‘vaccination group’, and higher in No vaccination group compared to Vaccination group **(D)**. Asterix shows significance between Omicron groups, and significance between healthy control and COVID-19 groups left out. *p*-value are from 2-way ANOVA with Tukey’s multiple comparisons test. **p < 0.01, ****p < 0.0001. Median, middle bar inside the box; IQR, 50% of the data; whiskers, 1.5 times the IQR.

To understand the impact of prior vaccination or prior infection on the genomic immune response to Omicron infection, we investigated the immune transcriptome (**Figure 3**; **Table S1**). These data sets were also compared to a naïve reference population (no previous SARS-CoV-2 infection and no vaccination) (10) and to hospitalized patients that had been infected with the Alpha variant (7). Bulk RNA-seq on buffy coats isolated within the first two days after validated Omicron infection was performed with an average sequencing depth of 200 million reads per sample. Because no day 1-3 samples were available for the prior infection/no vaccination group (**Figure 1**), the analysis was limited to the no prior infection/no vaccination, no prior infection/vaccination and prior infection/no vaccination groups. First, we directly compared the transcriptomes of the vaccinated and unvaccinated Omicron cohorts without prior infection with a reference cohort of 30 healthy individuals from the same geographic area (Tyrol Control Transcriptomes, TCT). Expression of 489 and 732 genes was induced significantly in the no vaccination and vaccination group, respectively. Expression of 146 and 246 genes was reduced. Next, we compared the transcriptomes between TCT and no vaccination/prior infection group and found 356 significantly induced and 153 reduced genes. GSEA analyses linked the induced genes in three groups to innate immune responses including interferon response and cytokine signaling through the JAK/STAT pathway (**Figures 3A, B**). Among the JAK/STAT responsive genes activated in the Omicron patients is the interferon induced family (IFI) and the antiviral OAS genes (**Figure 3C**). Overall, the differences observed between the cohorts were of a quantitative rather than a qualitative nature.

We directly explored those genes that were differentially expressed between vaccinated and unvaccinated outpatients depending on their prior infection status. (**Figures 3C, D**, **Table S1**). In general, the activation of genes linked to antiviral defense programs and COVID-19 disease predictors was lowest in the vaccinated patients without prior infection and highest in the patients infected with the Alpha variant (**Figures 3C, D**). The antiviral defense programs encompass interferon-induced gene families, including the OAS family that counteract viral attacks by degrading viral RNAs. We also identified genes that had not previously linked to COVID-19, such as USP18, an IFN-induced gene encoding a negative regulator of type I IFN signaling (11), which highly activated in unvaccinated Omicron patients and Alpha patients.

Of particular interest is the OAS family of antiviral enzymes where hypomorphic mutations have been associated with susceptibility to viral infection and activating mutations with autoimmune disease. The OAS1 member of this family is of particular interest as it harbors a mutation traced back to the Neanderthal genome that results in a splice variant associated with protection from severe COVID-19 (12). RNA-seq data revealed that the rs10774671 haplotype was found in 83% of our control population (TCT cohort), 86% and 85% in the unvaccinated and vaccinated Omicron population, respectively and 96% of the Alpha population. Induction of OAS1 expression is highest in unvaccinated Omicron patients and the Alpha patients (**Figure 3C**).

## Discussion

Omicron infection of unvaccinated and vaccinated people has been reported to result in milder disease relative to previous variants (13). However, unlike the response to Delta breakthrough infection (5, 6, 14), Omicron breakthrough infections might result in lower levels of neutralizing antibodies (15). The muted antibody response may be due to the high share of asymptomatic and mild infections as is also indicated by a less active immune transcriptome shown in our study. Notably, individuals with prior vaccination showed significant Omicron neutralization activity, even in the presence of a blunted transcriptional response. Naïve individuals demonstrated significantly higher transcriptional response but a less robust humoral response. The response in vaccinated or prior infection groups was quantitatively less, but similar in unvaccinated/no prior infection group, when compared to the transcriptional response of hospitalized individuals with Alpha infection. Vaccination was vastly superior to prior infection in promoting neutralizing Omicron antibodies and subsequent Omicron infection yielded an elevated antibody response in both groups. In contrast, Omicron infection in the antigen naïve population did not result in any significant antibody production within a time window of two weeks, suggesting that the initial exposure to Omicron spike proteins does not elicit a substantial immune response.

The study documented that both antibody and immune transcriptional responses to Omicron infection are modified by prior vaccination more significantly than by prior infection. Antibody response is higher in concert with a blunted immune transcriptional response. In this group of largely previously healthy individuals there were no significant differences in symptomatology that correlated with either antibody or transcriptional response, but it needs to be noted that few patients in the cohorts developed severe disease and all were outpatients. It is established that prior vaccination is protective against hospitalization for Omicron infection (16) and known that antibody generation is impacted by antigen exposure history and presentation through vaccination as compared to infection (17). While other studies have similarly examined antibody titers and neutralizing responses in Omicron breakthrough infections, here we integrated the innate immune transcriptome response. This added information layer enabled us to reveal that vaccination, but not previous infection, blunted the Omicron-induced immune transcriptome response at the same time it augmented the antibody response. There are relatively few studies comparing transcriptome response following SARS-CoV-2 infection in vaccinated and unvaccinated individuals. In a previous study, we examined the comparative response with vaccination prior to infection and found an enhanced JAK-STAT-mediated response in vaccinated as compared to unvaccinated patients (8). There are three significant differences between this previous study and the present one. First, here we examined the response a mean of 111 days following vaccination whereas in the previous study it was only 11 days. Second, here all patients were outpatients while in the previous study all patients were hospitalized with infection. Third, in the previous study patients were infected with the SARS-CoV-2 Beta variant while in the present study we examined the transcriptional response to Omicron. We found an important difference, while JAK-STAT signaling was enhanced in the vaccinated patients in the previous study, here we found a blunted response. It is possible this is due to a difference in disease severity or the timing of the vaccination prior to infection or a variant-specific because breakthrough Omicron infection has been reported to be less immunogenic than infection with other variants (15, 18, 19). Other groups as well as we have examined the immune transcriptional response post vaccination (10, 20–22) and document that, like acute SARS-CoV-2 infection, innate immunity pathways are activated.

The role of prior vaccination in augmenting antibody response to Omicron infection has been reported across a range of available vaccines but not all studies examined the response following Omicron infection. Here we specifically focused on the response following documented Omicron infection. A study available in preprint form similarly reports comparing 14 vaccinated and 7 unvaccinated Omicron infected patients show higher convalescent titers and neutralization activity than unvaccinated patients (23). Omicron-infected patients with prior vaccination with both Pfizer-BNT162b2 or J&J-Ad26 were shown to develop higher Delta virus neutralization titers (24). Broader T cell immunity following Omicron infection in Pfizer/BioNTech BNT162 vaccinated individuals is reported (25).

Higher neutralization activity against Omicron was found in Ad26.COV2.S vaccinated individuals who then experienced breakthrough Delta infection (26, 27). Prior Comirnaty or Coronvac vaccination results in higher neutralization titers against Omicron subvariants as well as other SARS-CoV-2 variants (28). The ability of Omicron to evade the immune system is both an individual clinical concern as well as an issue for population-based infection control. Breakthrough Omicron infections are documented in individuals with a range of vaccine-induced protective antibody titers (29). Booster vaccination can increase antibody levels and activity but cannot absolutely prevent breakthrough infection (30). Overall, it has been observed that Omicron is the most neutralization resistant SARS-CoV-2 variant of concern (VOC) (31).

A recent study determined that neutralizing antibody titers tracked with clinical severity of Omicron breakthrough infections in previously vaccinated patients (15). We did not see any significant differences in antibody levels between asymptomatic to mild breakthrough infections compared to moderate or severe infections. These differences might lie in the demographics of the two populations. Our cohort was significantly younger with few underlying health conditions and milder disease. We also did not observe a correlation between antibody levels and disease severity in Omicron breakthrough infections of patients that had previous documented infections by other SARS-CoV-2 variants. Interferon type I IFN (IFN-I) signaling has been previously reported to correlate with higher disease severity in a study of five unvaccinated patients admitted to hospital with the ancestral COVID-19 strain (32, 33) and correlated with progression to pulmonary fibrosis in a study of 12 unvaccinated hospitalized severely ill patients infected with the ancestral strain (34). In our larger study of outpatients infected with Omicron the blunted interferon gamma response found in vaccinated individuals was not correlated with more severe disease. The SARS-CoV-2 pandemic has been typified by progression through evolving variants. Our study recruited patients during the initial BA.1 phase of Omicron infection. It has been reported that neutralization evasion is similar for the different Omicron variants (35), suggesting that results reported here may be predictive of response to the newer variants as well.

Identification of biomarkers predictive of disease progression has the possibility of contributing positively to clinical management of patients. Type I Interferon response, RIG-I signaling, and multiple proteins known to be induced by interferon signaling including CXCL10 (also known as Interferon Gamma-induced protein, IP-10), MCP-1, MCP-2 and CXCL11 have been reported in a recent preprint to be associated with COVID-19 disease progression in a study of eight patients (22). As discussed above, in the cohort study here we had insufficient numbers of patients with progressive severe disease to test for biomarkers of progression. We did, however, note that CXCL10, identified as a marker of disease progression in this cited study was significantly elevated in the unvaccinated as compared to vaccinated patients in our study. We also found significant elevations in CCL2 and USP18. CCL2 has been linked to influenza induced disease severity in an animal model (36) and documented as one of the significantly increased chemokines in severe COVID-19 hyperinflammation syndrome (37). USP18 is capable of attenuating Type 1 Interferon signaling, which has been associated with persistence of both Hepatitis B and C infection in liver (38). It is possible that elevations of USP18 might contribute to SARS-CoV-2 persistence as well, an aspect of SARS-CoV-2 infection that was not examined in this study but could be approached in a systematic way in a prospective study designed around the question of SARS-CoV-2 viral persistence.

In summary, vaccination prior to Omicron infection modifies humoral and transcriptional responses with higher antibody and neutralization titers and lower interferon gamma activation than that found in unvaccinated individuals either with or without prior infection. Transcriptome studies drew attention to specific genes of interest for future studies of the impact of vaccination on SARS-CoV-2 disease presentation and progression.

## Methods

### Ethics

This study was approved (EK Nr: 1064/2021) by the Institutional Review Board (IRB) of the Office of Research Oversight/Regulatory Affairs, Medical University of Innsbruck, Austria, which is responsible for all human research studies conducted in the State of Tyrol (Austria). The investigators do not need to have an affiliation with the University of Innsbruck. Participant information was coded and anonymized.

### Study Population, Study Design and Recruitment

A total of 83 patients infected with Omicron were recruited for the study under informed consent. Thirty-four with no history of prior vaccination or prior infection with other SARS-CoV-2 variants, 23 patients who had received 2 or 3 doses of the BNT162b2 vaccine, 19 unvaccinated patients with prior infections and 7 vaccinated patients with prior infection (**Table 1**). Recruitment and blood sample collection took place between December 2021 and March 2022. Patients were enrolled after either attending an outpatient clinic at Krankenhaus St. Vinzenz Zams for SARS-CoV-2 PCR testing, or, as a family contact with a positive SARS-CoV-2 PCR test. Variant PCR screening for single nucleotide polymorphisms characteristic for Omicron was used to detect Omicron infection. Symptomatic patients presented themselves to the clinic within a few days of initial symptoms. Single or serial blood samples were collected from consenting patients. Day numbers for samples refers to the number of days following initial positive SARS-CoV-2 RT-PCR test. Patient recruitment was performed by a medic who assessed clinical status including performance of an oxygen saturation (SpO2) test. The clinical spectrum of the patients’ SARS-CoV-2 symptoms were classified based on the National Institutes of Health (NIH) treatment guidelines (https://files.covid19treatmentguidelines.nih.gov/guidelines/section/section_43.pdf). A subset of patients across groups reported subjective shortness of breath (SOB) but did not show clinical evidence of lower respiratory disease during clinical assessment or by imaging. These patients were classified as mild with shortness of breath for this study. Information on prior infection was obtained from medical records. No patient was admitted to hospital with their prior infection. Disease severity with the prior infection in these patients ranged from mild to moderate to severe based on the NIH guidelines. Prior infection variant and number of days (mean and range) infected prior to Omicron infection for the no vaccination/prior infection group are as follows: Delta (n=9, mean 79 days, range 42-189 days, D614G (n=4, mean 181 days, range 55-289 days, unknown variant n=6, mean 70 days, range 21-158 days. Prior infection variant and number of days (mean and range) infected prior to Omicron infection for the vaccination/prior infection group are as follows: Delta (n=4, mean 55 days, range 34-64 days, D614G (n=2, mean 181 days, range 67-295 days, Alpha n=1, 303 days. The reference “Alpha” cohort consists of individuals (n=36, mean age 71 years) infected with the Alpha variant in the spring of 2021 that were hospitalized after developing COVID-19 (7). Blood samples were collected within 10 days of verified SARS-CoV-2 infection and the RNA was prepared by the same person, who isolated the RNA for the current Omicron study. RNA-seq was conducted using the same supplies and equipment as in the current Omicron study and data analysis was performed by the same person (HKL). A waiver of informed consent was obtained from the Institutional Review Board (IRB) of the Office of Research Oversight/Regulatory Affairs, Medical University of Innsbruck (https://www.i-med.ac.at/ethikkommission/). Written informed consent was obtained from all subjects. This study was determined to impose minimal risk on participants. All methods were carried out in accordance with relevant guidelines and regulations. All research has been have been performed in accordance with the Declaration of Helsinki (https://www.wma.net/policies-post/wma-declaration-of-helsinki-ethical-principles-for-medical-research-involving-human-subjects/). In addition, we followed the ‘Sex and Gender Equity in Research – SAGER – guidelines’ and included sex and gender considerations where relevant.

### Antibody Assay

Whole blood was collected by medical personnel after subjects had tested positive for SARS-CoV-2. Antibody containing sera were obtained by centrifuging EDTA blood samples for 10 min at 4,000g. End-point binding IgG antibody titers to various SARS-CoV-2–derived antigens were measured using the Meso Scale Discovery (MSD) platform. SARS-CoV-2 spike, nucleocapsid, Alpha, Beta, Gamma, Delta, and Omicron spike subdomains were assayed using the V-plex multispot COVID-19 serology kits (Panel 23 (IgG) Kit, K15567U). Plates were coated with the specific antigen on spots in the 96 well plate and the bound antibodies in the samples (1:50000 dilution) were then detected by anti-human IgG antibodies conjugated with the MSD SULPHO-TAG which is then read on the MSD instrument which measures the light emitted from the tag.

### ACE2 Binding Inhibition (Neutralization) ELISA

The V-PLEX COVID-19 ACE2 Neutralization kit (Meso Scale Discovery, Panel 23 (ACE2) Kit, K15570U) was used to quantitatively measure antibodies that block the binding of ACE2 to its cognate ligands (SARS-CoV-2 and variant spike subdomains). Plates were coated with the specific antigen on spots in the 96 well plate and the bound antibodies in the samples (1:10 dilution) were then detected by Human ACE2 protein conjugated with the MSD SULPHO-TAG which is then read on the MSD instrument which measures the light emitted from the tag.

### Extraction of the Buffy Coat and Purification of RNA

Whole blood was collected, and total RNA was extracted from the buffy coat and purified using the Maxwell RSC simply RNA Blood Kit (Promega) according to the manufacturer’s instructions. The concentration and quality of RNA were assessed by an Agilent Bioanalyzer 2100 (Agilent Technologies, CA).

### mRNA Sequencing (mRNA-Seq) and Data Analysis

The Poly-A containing mRNA was purified by poly-T oligo hybridization from 1 mg of total RNA and cDNA was synthesized using SuperScript III (Invitrogen, MA). Libraries for sequencing were prepared according to the manufacturer’s instructions with TruSeq Stranded mRNA Library Prep Kit (Illumina, CA, RS-20020595) and paired-end sequencing was done with a NovaSeq 6000 instrument (Illumina) yielding 200-350 million reads per sample.

The raw data were subjected to QC analyses using the FastQC tool (version 0.11.9) (https://www.bioinformatics.babraham.ac.uk/projects/fastqc/). mRNA-seq read quality control was done using Trimmomatic (39) (version 0.36) and STAR RNA-seq (40) (version STAR 2.5.4a) using 150 bp paired-end mode was used to align the reads (hg19). HTSeq (41) (version 0.9.1) was to retrieve the raw counts and subsequently, Bioconductor package DESeq2 (42) in R (https://www.R-project.org/) was used to normalize the counts across samples (43) and perform differential expression gene analysis. Additionally, the RUVSeq (44) package was applied to remove confounding factors. The data were pre-filtered keeping only genes with at least ten reads in total. The visualization was done using dplyr (https://CRAN.R-project.org/package=dplyr) and ggplot2 (45). The genes with log2 fold change >1 or <-1 and adjusted p-value (pAdj) <0.05 corrected for multiple testing using the Benjamini-Hochberg method were considered significant and then conducted gene enrichment analysis (GSEA, https://www.gsea-msigdb.org/gsea/msigdb).

### Quantification and Statistical Analysis

Differential expression gene (DEG) identification used Bioconductor package DESeq2 in R. P-values were calculated using a paired, two-side Wilcoxon test and adjusted p-value (pAdj) corrected using the Benjamini–Hochberg method. The cut-off value for the false discovery rate was pAdj > 0.05. Genes with log_2_ fold change >1 or <-1, pAdj <0.05 and without 0 value from all sample were considered significant. For significance of each GSEA category, significantly regulated gene sets were evaluated with the Kolmogorov-Smirnov statistic. P-values of antibody between two groups were calculated using one-tailed Mann-Whitney t-test on GraphPad Prism software (version 9.0.0). A value of *P < 0.05, **P < 0.01, ***P < 0.001, ****P < 0.0001 was considered statistically significant.

## Data Availability Statement

The datasets presented in this study can be found in online repositories. The names of the repository/repositories and accession number(s) can be found in the article/**Supplementary Material**. RNA-seq data generated from this study were deposited under the accession GSE201530 in the Gene Expression Omnibus (GEO). RNA-seq data from buffy coat of healthy control and COVID-19 Alpha patients were obtained GSE189039, GSE190747 and GSE190680.

## Ethics Statement

This study was approved (EK Nr: 1064/2021) by the Institutional Review Board (IRB) of the Office of Research Oversight/Regulatory Affairs, Medical University of Innsbruck, Austria, which is responsible for all human research studies conducted in the State of Tyrol (Austria). Written informed consent to participate in this study was provided by the participants’ legal guardian/next of kin.

## Author Contributions

HL, LK, PF, and LH designed the study. LK Sr, MF, YC, CJ, PK, MO, CB, NK, AZ, and GW recruited patients and collected material. HL analyzed RNA-seq data. MW and YD conducted IgG antibody and neutralization assay. HL, PF, and LH analyzed data. HL administrated project. HL, PF, and LH wrote the paper. All authors read and approved the manuscript.

## Funding

This work was supported by the Intramural Research Program (IRP) of the National Institute of Diabetes and Digestive and Kidney Diseases (NIDDK).

## Conflict of Interest

Authors LK, MF, YC, CJ, and PK are employed by TyrolPath Obrist Brunhuber GmbH, Zams, Austria.

The remaining authors declare that the research was conducted in the absence of any commercial or financial relationships that could be construed as a potential conflict of interest.

## Publisher’s Note

All claims expressed in this article are solely those of the authors and do not necessarily represent those of their affiliated organizations, or those of the publisher, the editors and the reviewers. Any product that may be evaluated in this article, or claim that may be made by its manufacturer, is not guaranteed or endorsed by the publisher.

## References

[1] Flemming A. Omicron, the Great Escape Artist. Nat Rev Immunol (2022) 22:75. doi: 10.1038/s41577-022-00676-6 35017722 PMC8749340

[2] VanBlargan LA Errico JM Halfmann PJ Zost SJ Crowe JE Jr. Purcell LA. An Infectious SARS-CoV-2 B.1.1.529 Omicron Virus Escapes Neutralization by Therapeutic Monoclonal Antibodies. Nat Med (2022) 28:490–5. doi: 10.1038/s41591-021-01678-y PMC8767531 35046573

[3] Chatterjee D Tauzin A Marchitto L Gong SY Boutin M Bourassa C. SARS-CoV-2 Omicron Spike Recognition by Plasma From Individuals Receiving BNT162b2 mRNA Vaccination With a 16-Week Interval Between Doses. Cell Rep (2022) 38:110429. doi: 10.1016/j.celrep.2022.110429 35216664 PMC8823958

[4] Edara VV Manning KE Ellis M Lai L Moore KM Foster SL. mRNA-1273 and BNT162b2 mRNA Vaccines Have Reduced Neutralizing Activity Against the SARS-CoV-2 Omicron Variant. Cell Rep Med (2022) 3:100529. doi: 10.1016/j.xcrm.2022.100529 35233550 PMC8784612

[5] Walls AC Sprouse KR Bowen JE Joshi A Franko N Navarro MJ. SARS-CoV-2 Breakthrough Infections Elicit Potent, Broad, and Durable Neutralizing Antibody Responses. Cell (2022) 185:872–80.e3. doi: 10.1016/j.cell.2022.01.011 35123650 PMC8769922

[6] Collier AY Brown CM McMahan KA Yu J Liu J Jacob-Dolan C. Characterization of Immune Responses in Fully Vaccinated Individuals Following Breakthrough Infection With the SARS-CoV-2 Delta Variant. Sci Transl Med (2022) 7(5):e155944. doi: 10.1126/scitranslmed.abn6150 PMC8995036 35258323

[7] Lee HK Knabl L Knabl L Wieser M Mur A Zabernigg A. Immune Transcriptome Analysis of COVID-19 Patients Infected With SARS-CoV-2 Variants Carrying the E484K Escape Mutation Identifies a Distinct Gene Module. Sci Rep (2022) 12:2784. doi: 10.1038/s41598-022-06752-0 35181735 PMC8857234

[8] Knabl L Lee HK Wieser M Mur A Zabernigg A Knabl L. BNT162b2 Vaccination Enhances Interferon-JAK-STAT-Regulated Antiviral Programs in COVID-19 Patients Infected With the SARS-CoV-2 Beta Variant. Commun Med (2022) 2:17. doi: 10.1038/s43856-022-00083-x 35465056 PMC9029844

[9] Neghaiwi BH. Austria Reports First Suspected Case of Omicron COVID-19 Variant (Reuters, November 28, 2021).

[10] Lee HK Knabl L Moliva JI Knabl L Werner AP Boyoglu-Barnum S. mRNA Vaccination in Octogenarians 15 and 20 Months After Recovery From COVID-19 Elicits Robust Immune and Antibody Responses That Include Omicron. Cell Rep (2022) 39(2):110680. doi: 10.1016/j.celrep.2022.110680 35395191 PMC8947943

[11] Basters A Knobeloch KP Fritz G. USP18 - a Multifunctional Component in the Interferon Response. Biosci Rep (2018) 38(6):BSR20180250. doi: 10.1042/BSR20180250 30126853 PMC6240716

[12] Huffman JE Butler-Laporte G Khan A Pairo-Castineira E Drivas TG Peloso GM. Multi-Ancestry Fine Mapping Implicates OAS1 Splicing in Risk of Severe COVID-19. Nat Genet (2022) 54:125–7. doi: 10.1038/s41588-021-00996-8 PMC8837537 35027740

[13] Wolter N Jassat W Walaza S Welch R Moultrie H Groome M. Early Assessment of the Clinical Severity of the SARS-CoV-2 Omicron Variant in South Africa: A Data Linkage Study. Lancet (2022) 399:437–46. doi: 10.1016/S0140-6736(22)00017-4 PMC8769664 35065011

[14] Wratil PR Stern M Priller A Willmann A Almanzar G Vogel E. Three Exposures to the Spike Protein of SARS-CoV-2 by Either Infection or Vaccination Elicit Superior Neutralizing Immunity to All Variants of Concern. Nat Med (2022) 28:496–503. doi: 10.1038/s41591-022-01715-4 35090165

[15] Servellita V Syed AM Morris MK Brazer N Saldhi P Garcia-Knight M. Neutralizing Immunity in Vaccine Breakthrough Infections From the SARS-CoV-2 Omicron and Delta Variants. Cell (2022) 185:1539–48.e5. doi: 10.1016/j.cell.2022.03.019 35429436 PMC8930394

[16] Nyberg T Ferguson NM Nash SG Webster HH Flaxman S Andrews N. Comparative Analysis of the Risks of Hospitalisation and Death Associated With SARS-CoV-2 Omicron (B.1.1.529) and Delta (B.1.617.2) Variants in England: A Cohort Study. Lancet (2022) 399:1303–12. doi: 10.1016/S0140-6736(22)00462-7 PMC8926413 35305296

[17] Röltgen K Nielsen SCA Silva O Younes SF Zaslavsky M Costales C. Immune Imprinting, Breadth of Variant Recognition, and Germinal Center Response in Human SARS-CoV-2 Infection and Vaccination. Cell (2022) 185:1025–40.e14. doi: 10.1016/j.cell.2022.01.018 35148837 PMC8786601

[18] Rössler A Knabl L von Laer D Kimpel J. Neutralization Profile After Recovery From SARS-CoV-2 Omicron Infection. N Engl J Med (2022) 386:1764–6. doi: 10.1056/NEJMc2201607 PMC9006769 35320661

[19] Rossler A Riepler L Bante D von Laer D Kimpel J. SARS-CoV-2 Omicron Variant Neutralization in Serum From Vaccinated and Convalescent Persons. N Engl J Med (2022) 386:698–700. doi: 10.1056/NEJMc2119236 35021005 PMC8781314

[20] Zhang Y Guo X Li C Kou Z Lin L Yao M. Transcriptome Analysis of Peripheral Blood Mononuclear Cells in SARS-CoV-2 Naïve and Recovered Individuals Vaccinated With Inactivated Vaccine. Front Cell Infect Microbiol (2021) 11:821828. doi: 10.3389/fcimb.2021.821828 35186784 PMC8851474

[21] Arunachalam PS Scott MKD Hagan T Li C Feng Y Wimmers F. Systems Vaccinology of the BNT162b2 mRNA Vaccine in Humans. Nature (2021) 596(7872):410–6. doi: 10.1038/s41586-021-03791-x PMC8761119 34252919

[22] Hu Z van der Ploeg K Chakraborty S Arunachalam P Mori D Jacobson K. Early Immune Responses Have Long-Term Associations With Clinical, Virologic, and Immunologic Outcomes in Patients With COVID-19. Res Sq (2022) rs.3.rs-847082. doi: 10.21203/rs.3.rs-847082/v1

[23] Seaman MS Siedner MJ Boucau J Lavine CL Ghantous F Liew MY. Vaccine Breakthrough Infection With the SARS-CoV-2 Delta or Omicron (BA.1) Variant Leads to Distinct Profiles of Neutralizing Antibody Responses. medRxiv (2022) 2022.03.02.22271731. doi: 10.1101/2022.03.02.22271731 PMC9675445 36214224

[24] Khan K Karim F Cele S Reedoy K San JE Lustig G. Omicron Infection Enhances Delta Antibody Immunity in Vaccinated Persons. Nature (2022). doi: 10.1038/s41586-022-04830-x PMC9279144 35523247

[25] Lang-Meli J Luxenburger H Wild K Karl V Oberhardt V Salimi Alizei E. SARS-CoV-2-Specific T-Cell Epitope Repertoire in Convalescent and mRNA-Vaccinated Individuals. Nat Microbiol (2022) 7:675–9. doi: 10.1038/s41564-022-01106-y PMC9064790 35484232

[26] Kitchin D Richardson SI van der Mescht MA Motlou T Mzindle N Moyo-Gwete T. Ad26.Cov2.S Breakthrough Infections Induce High Titers of Neutralizing Antibodies Against Omicron and Other SARS-CoV-2 Variants of Concern. Cell Rep Med (2022) 3:100535. doi: 10.1016/j.xcrm.2022.100535 35474744 PMC8828412

[27] Montefiori DC. Enhanced Immunity After Ad26.COV2.S Vaccine Breakthrough Infection. Cell Rep Med (2022) 3:100579. doi: 10.1016/j.xcrm.2022.100579 35474738 PMC8922435

[28] Cheng SM Mok CKP Chan KC Ng SS Lam BH Luk LL. SARS-CoV-2 Omicron Variant BA.2 Neutralisation in Sera of People With Comirnaty or CoronaVac Vaccination, Infection or Breakthrough Infection, Hong Kong, 2020 to 2022. Euro Surveill (2022) 27(18):2200178. doi: 10.2807/1560-7917.ES.2022.27.18.2200178 PMC9074393 35514306

[29] Adachi E Nagai E Saito M Isobe M Konuma T Koga M. Anti-Spike Protein Antibody Titer at the Time of Breakthrough Infection of SARS-CoV-2 Omicron. J Infect Chemother (2022) 28:1015–7. doi: 10.1016/j.jiac.2022.03.021 PMC8971116 35397976

[30] Woldemeskel BA Garliss CC Aytenfisu TY Johnston TS Beck EJ Dykema AG. SARS-CoV-2 -Specific Immune Responses in Boosted Vaccine Recipients With Breakthrough Infections During the Omicron Variant Surge. JCI Insight (2022) 7(10):e159474. doi: 10.1172/jci.insight.159474 PMC9220829 35389888

[31] Sievers BL Chakraborty S Xue Y Gelbart T Gonzalez JC Cassidy AG. Antibodies Elicited by SARS-CoV-2 Infection or mRNA Vaccines Have Reduced Neutralizing Activity Against Beta and Omicron Pseudoviruses. Sci Transl Med 14 (2022) 14(634):eabn7842. doi: 10.1126/scitranslmed.abn7842 PMC8891085 35025672

[32] Wang X Sanborn MA Dai Y Rehman J. Temporal Transcriptomic Analysis Using TrendCatcher Identifies Early and Persistent Neutrophil Activation in Severe COVID-19. JCI Insight (2022) 7(7):e157255. doi: 10.1172/jci.insight.157255 PMC9057597 35175937

[33] Zhu L Yang P Zhao Y Zhuang Z Wang Z Song R. Single-Cell Sequencing of Peripheral Mononuclear Cells Reveals Distinct Immune Response Landscapes of COVID-19 and Influenza Patients. Immunity (2020) 53:685–96.e3. doi: 10.1016/j.immuni.2020.07.009 32783921 PMC7368915

[34] Wang Z Zhang Y Yang R Wang Y Guo J Sun R. Landscape of Peripheral Blood Mononuclear Cells and Soluble Factors in Severe COVID-19 Patients With Pulmonary Fibrosis Development. Front Immunol (2022) 13:831194. doi: 10.3389/fimmu.2022.831194 35558069 PMC9088015

[35] Arora P Zhang L Rocha C Sidarovich A Kempf A Schulz S. Comparable Neutralisation Evasion of SARS-CoV-2 Omicron Subvariants BA.1, BA.2, and BA.3. Lancet Infect Dis (2022) 22(6):766–7. doi: 10.1016/S1473-3099(22)00224-9 PMC9005119 35427493

[36] Lai C Wang K Zhao Z Zhang L Gu H Yang P. C-C Motif Chemokine Ligand 2 (CCL2) Mediates Acute Lung Injury Induced by Lethal Influenza H7N9 Virus. Front Microbiol (2017) 8:587. doi: 10.3389/fmicb.2017.00587 28421067 PMC5379033

[37] Merad M Martin JC. Pathological Inflammation in Patients With COVID-19: A Key Role for Monocytes and Macrophages. Nat Rev Immunol (2020) 20:355–62. doi: 10.1038/s41577-020-0331-4 PMC7201395 32376901

[38] Kang JA Jeon YJ. Emerging Roles of USP18: From Biology to Pathophysiology. Int J Mol Sci 21 (2020) 21(18):6825. doi: 10.3390/ijms21186825 PMC7555095 32957626

[39] Bolger AM Lohse M Usadel B. Trimmomatic: A Flexible Trimmer for Illumina Sequence Data. Bioinformatics (2014) 30:2114–20. doi: 10.1093/bioinformatics/btu170 PMC4103590 24695404

[40] Dobin A Davis CA Schlesinger F Drenkow J Zaleski C Jha S. STAR: Ultrafast Universal RNA-Seq Aligner. Bioinformatics (2013) 29:15–21. doi: 10.1093/bioinformatics/bts635 23104886 PMC3530905

[41] Anders S Pyl PT Huber W. HTSeq–a Python Framework to Work With High-Throughput Sequencing Data. Bioinformatics (2015) 31:166–9. doi: 10.1093/bioinformatics/btu638 PMC4287950 25260700

[42] Love MI Huber W Anders S. Moderated Estimation of Fold Change and Dispersion for RNA-Seq Data With Deseq2. Genome Biol (2014) 15:550. doi: 10.1186/s13059-014-0550-8 25516281 PMC4302049

[43] Zhao Y Li MC Konate MM Chen L Das B Karlovich C. TPM, FPKM, or Normalized Counts? A Comparative Study of Quantification Measures for the Analysis of RNA-Seq Data From the NCI Patient-Derived Models Repository. J Transl Med (2021) 19:269. doi: 10.1186/s12967-021-02936-w 34158060 PMC8220791

[44] Risso D Ngai J Speed TP Dudoit S. Normalization of RNA-Seq Data Using Factor Analysis of Control Genes or Samples. Nat Biotechnol (2014) 32:896–902. doi: 10.1038/nbt.2931 25150836 PMC4404308

[45] Wickham H. Ggplot2 : Elegant Graphics for Data Analysis. New York: Springer (2009).

