# Supplementary material for: Prior Vaccination Exceeds Prior Infection in Eliciting Innate and Humoral Immune Responses in Omicron Infected Outpatients
Source: Front Immunol. 2022 Jun 15;13:916686. doi: 10.3389/fimmu.2022.916686 (PMC9240221; doi:10.3389/fimmu.2022.916686)
Supplement: Supplementary file 1 [file DataSheet_1.docx]

Supplementary Material

**Prior vaccination exceeds prior infection in eliciting innate and humoral immune responses in Omicron infected outpatients**

**Hye Kyung Lee^1,^*^,†^, Ludwig Knabl^2,^*^,†^, Mary Walter^3^, Ludwig Knabl Sr. ^4^, Yuhai Dai^3^, Magdalena Füßl^2^, Yasemin Caf^2^, Claudia Jeller^2^, Philipp Knabl^2^, Martina Obermoser^5^, Christof Baurecht^5^, Norbert Kaiser^5^, August Zabernigg^6^, Gernot M. Wurdinger^6^, Priscilla A. Furth^7,^*^,†^ and Lothar Hennighausen^1,^*^,†,a^**

^1^National Institute of Diabetes, Digestive and Kidney Diseases, National Institutes of Health, Bethesda, MD 20892, USA.

^2^TyrolPath Obrist Brunhuber GmbH, Zams, Austria.

^3^Clinical Core, National Institute of Diabetes, Digestive and Kidney Diseases, National Institutes of Health, Bethesda, MD 20892, USA.

^4^Division of Internal Medicine, Krankenhaus St. Vinzenz, Zams, Austria.

^5^Division of Internal Medicine, Krankenhaus St. Johann, St. Johann, Austria.

^6^Division of Internal Medicine, Krankenhaus Kufstein, Kufstein, Austria.

^7^Departments of Oncology & Medicine, Georgetown University, Washington, DC, USA.

^†^These authors contributed equally to this work.

^a^ This author has senior authorship.

*** Correspondence:**

HKL: [hyekyung.lee@nih.gov](mailto:hyekyung.lee@nih.gov); LK: [Ludwig.knabl@tyrolpath.at](mailto:Ludwig.knabl@tyrolpath.at); PAF: [paf3@georgetown.edu](mailto:paf3@georgetown.edu); LH: [lotharh@nih.gov](mailto:lotharh@nih.gov)

## Supplementary Figures

##
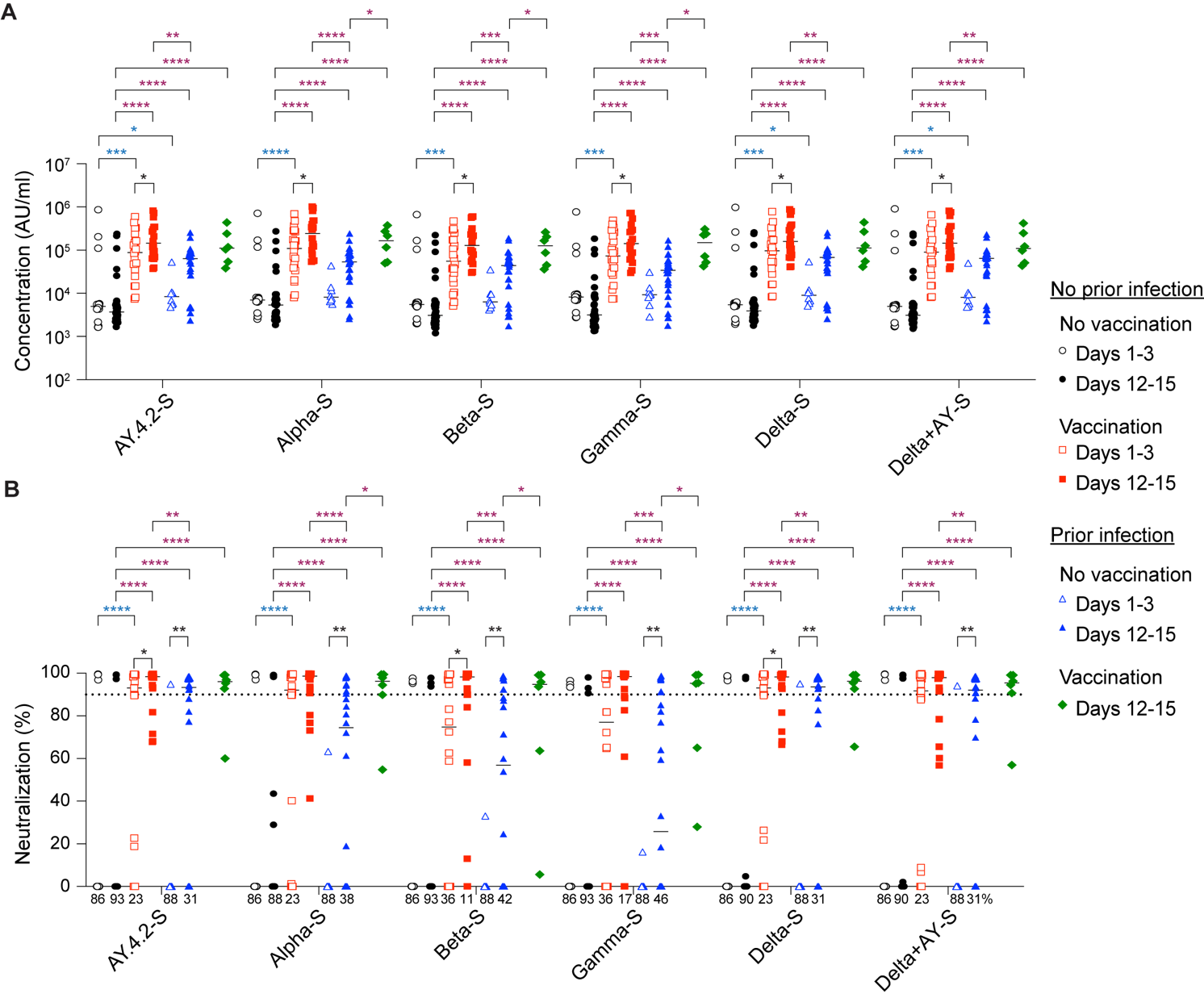


**Supplementary Figure 1.** **Antibody response of Omicron patients. (A)** Plasma IgG antibody binding the SARS-CoV-2 RBD (spike) from different strains in the no vaccinated and vaccinated Omicron patients without or with prior infection experience. (**B**) Neutralizing antibody response to virus spike protein of SARS-CoV-2 variants. *p*-value between two groups is from one-tailed Mann-Whitney t-test. Black Asterix shows significance between days in the same group, blue ones do significance of days 1-3 between groups, and purple ones do significance of days 12-15 between groups. **p* < 0.05, ***p* < 0.01, ****p* < 0, *****P* < 0.0001. Line at median.

**Supplementary tables**

**Supplementary Table 1.** List of significantly up-regulated genes in no vaccination cohort compared to vaccination cohort at Days1-2 after infection, log_2_ (fold change) and adjusted *p*-value.
